# Evolutionary Patterns in the Dentition of Duplicidentata (Mammalia) and a Novel Trend in the Molarization of Premolars

**DOI:** 10.1371/journal.pone.0012838

**Published:** 2010-09-20

**Authors:** Brian P. Kraatz, Jin Meng, Marcelo Weksler, Chuankui Li

**Affiliations:** 1 Department of Anatomy, Western University of Health Sciences, Pomona, California, United States of America; 2 Division of Paleontology, American Museum of Natural History, New York, New York, United States of America; 3 Department of Vertebrate Paleontology, Institute of Vertebrate Paleontology and Paleoanthropology, Beijing, China; University College London, United Kingdom

## Abstract

**Background:**

The cusp homology of Lagomorpha has long been problematic largely because their teeth are highly derived relative to their more typically tribosphenic ancestors. Within this context, the lagomorph central cusp has been particularly difficult to homologize with other tribosphenic cusps; authors have previously considered it the paracone, protocone, metacone, amphicone, or an entirely new cusp.

**Methodology/Principal Findings:**

Here we present newly described fossil duplicidentates (Lagomorpha and Mimotonidae) in the context of a well-constrained phylogeny to establish a nomenclatural system for cusps based on the tribosphenic pattern. We show that the central cusp of lagomorphs is homologous with the metaconule of other mammals. We also show that the buccal acquisition of a second cusp on the premolars (molarization) within duplicidentates is atypical with respect to other mammalian lineages; within the earliest lagomorphs, a second buccal cusp is added mesially to an isolated buccal cusp.

**Conclusions/Significance:**

The distal shift of the ‘ancestral’ paracone within early duplicidentates amounts to the changing of a paracone into a metacone in these lineages. For this reason, we support a strictly topological approach to cusp names, and suggest a discontinuity in nomenclature to capture the complexity of the interplay between evolutionary history and the developmental process that have produced cusp patterns in duplicidentates.

## Introduction

Understanding cusp homology among mammals is integral to deciphering their evolutionary history. It is often difficult, however, to homologize cusps in taxa that are highly derived; too often remnants of a primitive tribosphenic pattern have been overprinted by millions of years of evolution. Lagomorphs (rabbits, hares, and pikas) represent such a case, and there has been considerable debate over the last century as to how the cusps of lagomorph teeth relate to the tribosphenic condition. Much of the discussion has revolved around a prominent central cusp that appears in the upper cheek teeth of fossil taxa, and for an ontogenetically brief period, in extant lagomorphs. The homology of that cusp has been viewed differently by many workers, and has, at times, been considered to be homologous to the paracone [1]–[6], amphicone [7], metacone [8]–[11], or considered an evolutionary novelty [12]. This confusion has been exacerbated because the ancestral stock from which lagomorphs likely evolved has remained unclear, and in turn, tracing the evolution of lagomorph teeth from a more typically tribosphenic ancestor has been difficult.

**Figure 1:**
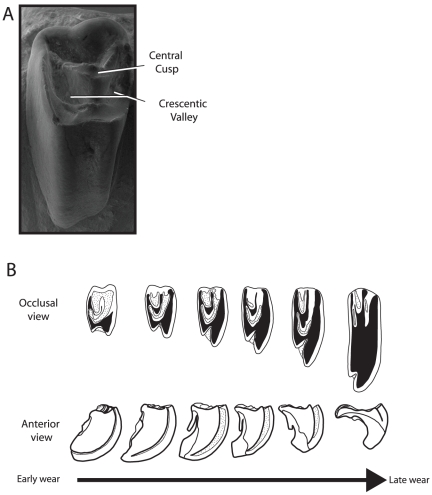
Images of upper cheek teeth of fossil lagomorphs that illustrate important crown structures and their changes with wear. A, SEM of a nearly unworn P^4^ of *Desmatolagus gobiensis* (AMNH 83703) showing the presence of the central cusp and crescentic valley. Buccal to the top, mesial to the left. B, various wear stages of P^4^ of *Hesperolagomys* n. sp., modified with permission from Bair [18], that illustrate the profound changes in crown morphology during the life of an animal. The top row shows occlusal views, buccal to top, mesial to left. The bottom row shows mesial views, buccal to left. White  =  enamel, black  =  dentine, and textured  =  cementum.

Understanding tooth cusp patterns within Lagomorpha is further complicated as both the upper and lower teeth of extant lagomorphs exhibit simple bilophodont morphology. The enamel surfaces of crowns are quickly worn away and are exposed as anterior and posterior lophs that are essentially enamel columns filled with dentine. This early crown wear erases cuspate structures, making the occlusal surface featureless. Despite this, both the central cusp and crescentic valley have long been recognized as prominent structures in fossil lagomorph teeth (Fig. 1, but see [8] and [10], among others). In simplest terms, fossil lagomorph teeth exhibit a dominant central cusp (literally, centered lingual–buccally and mesial–distally) that is bordered lingually by a prominent enamel v-shaped valley (i.e. crescentic valley), and buccally by a similar, but smaller valley. The apex of the crescentic valley points lingually, while two prominent arms are present that project mesio- and distobuccally. The crescentic valley, often filled with cementum, persists in many taxa after the enamel surface of the crown has worn away. The degree to which the structure persists within the tooth column (i.e. the depth of the crescentic valley) varies significantly within Lagomorpha, and a pattern of decreasing persistence (i.e. decreasing valley depth) over evolutionary time is apparent from the fossil record [13]. Several authors have claimed the crescent disappears in certain lineages, particularly within extant taxa, but detailed study of unworn extant teeth [7], [14], histological sections of developing teeth [15], and our own observations show that both the central cusp and crescentic valley can be found in all living taxa, albeit for a brief ontogenetic period. In short, the crescentic valley does not disappear in any taxa; it simply becomes less persistent. As prominent structures, the central cusp and crescentic valley should serve as landmarks to understand the overall cusp homology of lagomorphs, but it has been difficult to homologized them with tribosphenic structures, as supported by ([10]:34)

*“The details which are of interest in this connection* [understanding cusp homologies within Lagomorpha] *can be seen only on very little worn teeth, and before a great number of such teeth of different species are available for study an attempt to a definite interpretation of the cusps according to what has been said above might only give rise to further confusion*.”

Additionally, hypseledonty plays a dramatic role in the evolution of duplicidentate teeth (lagomorphs and their immediate ancestors, the mimotonids) and complicates the use of the central cusp and crescentic valley as landmark structures. As is typical for brachydont mammals, the upper teeth of the earliest duplicidentates have an enlarged lingual root as compared to two smaller buccal roots. In the earliest duplicidentate taxa, however, increased crown heights occur largely on the lingual side of the tooth, as the buccal side of the crown remains low-crowned. This condition is accentuated in subsequent taxa and has been termed unilateral hypsodonty. The result is that in many stem lagomorphs the lingual root never closes. The lingual portion of the tooth becomes ever growing while the buccal portion remains low-crowned. Over the life of the animal this causes substantial tooth rotation in the coronal plane, and in turn, dramatically changes the shape of the occlusal surface during the life of the animal (Fig. 1). Many workers have recognized these life history patterns (e.g., [16]–[17]), and more recently, a mathematical model was developed to illustrate the rotation of the tooth and its influence on the occlusal tooth pattern and shape [18]. The effect of this growth over the life of an animal is that lingual portion of the occlusal surface becomes greatly expanded via wear, and skews the relative positions of several prominent occlusal features, including the central cusp and crescentic valley. For all the reasons just outlined, it is imperative that any study focused on homologizing tooth cusps in duplicidentates use unworn or slightly worn teeth that not only allows for primary crown features to be observed, but also for various features to be understood in the their ‘original’ positions before differential crown wear occurs and distorts these data.

**Figure 2 pone-0012838-g002:**
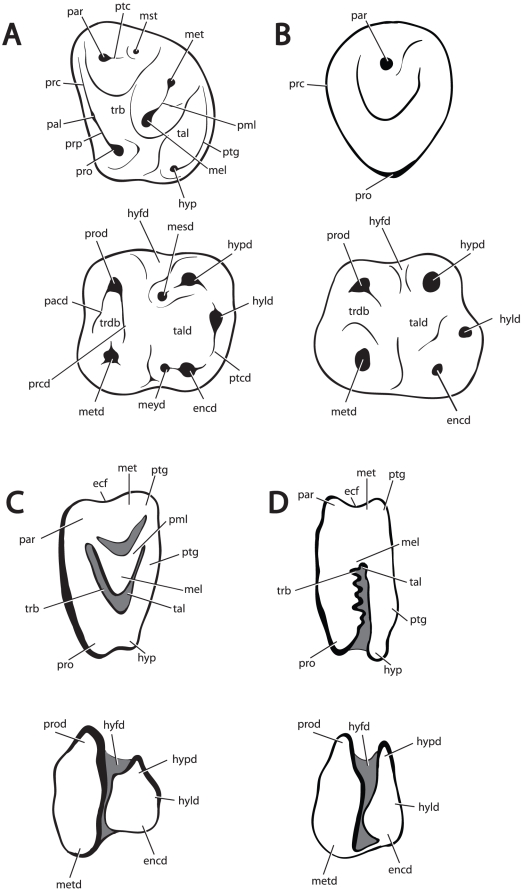
Tribosphenic terminology proposed in this study for crown structures of Duplicidentata. All teeth, buccal to top, mesial to left. A, Upper and lower molar of *Gomphos*. B, Upper and lower premolars of *Gomphos*. C, Upper and lower molars of *Desmatolagus*. D, Upper and lower molars for *Lepus*. These schematics should serve as guides for mimotonids (A & B), stem lagomorphs (C), and crown lagomorphs (D). Terminology follows [47], and includes; **ecf**, ectoflexus; **encd**, entoconid; **hyfd**, hypoflexid; **hyld**, hypoconulid; **hyp**, hypocone; **hypd**, hypoconid; **mel**, metaconule; **mesd**, mesoconid; **met**, metacone; **metd**, metaconid; **meyd**, mesostylid; **mst**, mesostyle; **pacd**, paracristid, **pal**, paraconule; **par**, paracone; **pml**, premetaconule crista; **prc**, preparaconule crista; **prcd**, protocristid; **pro**, protocone; **prod**, protoconid; **prp**, preprotocrista; **ptc**, postparacrista; **ptcd**, postcristid; **ptg**, postcingulum; **tal**, talon; **tald**, talonid; **trb**, trigon basin; and **trdb**, trigonid basin.

The goal of this paper is to present a nomenclature for tooth cusps of all living and extinct duplicidentates, based on the tribosphenic pattern. The central aspect of this work is the use of newly described material of an exceptionally preserved mimotonid, *Gomphos elkema*, and stem-lagomorph, *Dawsonolagus antiquus*, that show clear tribosphenic patterning, as well as clear relationships to crown lagomorphs [19]. We will use data from occlusion and wear facets to evaluate a hypothesis of cusp homology. We propose that the central cusp of lagomorphs is homologous with the tribosphenic metaconule, as well as apply tribosphenic terminology to all Duplicidentata crown structures (Fig. 2). Only Van Valen [20] has suggested that the central cusp of lagomorphs was the metaconule previously, and only tentatively, while recognizing the need for more corroborative evidence. Here were present that evidence in the form of 1) a tribosphenic nomenclatural system for mimotonids based on gross cusp topology and occlusal relationships, 2) a phylogenetic framework for duplicidentates that shows a clear ancestral-descendent relationship between mimotonids and lagomorphs, and finally, 3) extrapolations of the tribosphenic patterns recognized within mimotonids to stem- and crown-lagomorphs couched within this phylogenetic framework.

Central to this problem, however, is an understanding of how we recognize homology. This is a complicated question, with a long and well-documented history (see [21], for a thorough overview), and certainly beyond the scope of this paper to summarize the entire breadth of these discussions. We recognize here, therefore, that homology is *defined* as similarity due to common ancestry. To *diagnose* homology, however, one must first demonstrate that the structures in question are similar in form, position, and development. We suggest that these criteria are much more attainable now, and more specifically, we use the fossil record to trace the evolutionary history of various cusps among duplicidentates. Recently discovered primitive duplicidentate taxa have teeth that clearly show a tribosphenic cusp pattern, and these taxa help to establish a well-supported duplicidentate phylogeny [19], [22]. We also recognize that there is a disjunct between nomenclature and homology with respect to mammalian tooth cusps. We argue that this perspective is necessary, and that, at times, nomenclature does not (nor should) reflect homology. Finally, we recognize that a comprehensive treatment that includes crown lagomorphs is needed to apply tribosphenic nomenclature more broadly; this is our goal.

### History of Duplicidentate Cusp Nomenclature

While many authors have interpreted the central cusp of lagomorphs differently, it is an over simplification to merely list their preferred ‘homology statements’ as they were almost always made within the context of extensive discussion. Most often authors would not explicitly state which tribosphenic cusp they inferred the central cusp to be (e.g., [3], [10]), but rather, a likely homology was framed within various scenarios of the evolutionary origins of Lagomorpha. In this context, it is important to understand the basis upon which previous authors have inferred homology and denoted names to recognize how the evidence used to support these hypotheses (e.g., lagomorph ancestry) has often changed over the last century. We also note that the earlier tritubercular/tubercular-sectorial dentition patterns discussed in the Cope-Osborn theory were later renamed tribosphenic patterns *sensu* Simpson [23].

Major [3] conducted the first substantial systematic treatment of lagomorphs, and within that monograph he spent considerable time describing the teeth and cusps of fossil and extinct taxa. While the tritubercular/tubercular-sectorial theory (i.e. tribosphenic) had been largely established by this time, Major [3] rarely made tritubercular homology statements regarding specific cusps; instead he most often referred to occlusal structures in his own alphanumerical nomenclatural system. This is partially a product of his documented disagreement with some foundations of the Cope-Osborn theory [24], [25], and therefore, Major [3] did not actually specify with which primitive cusp he considered the central cusp of lagomorph to be homologous. Major's perspective on the central cusp was largely influenced by his hypothesis that lagomorphs' ancestral stock came from within a *Pelycodus – Plesiadapis* group [3], [25]. While he is explicit in this designation, and goes as far as to figure both *Pelycodus* and *Plesiadapis* upper molars to compare with an upper molar of *Caprolagus* ([3]; plate 36, figs. 1–3), he does not use tritubercular terminology. This approach is interesting in that Major [3], [25], at times, used tritubercular terms for lower teeth, yet not for upper teeth. Regardless, Major [3] did denote the central cusp in lagomorphs as ‘6,’ and similarly denotes a cusp that would now be considered the protocone as ‘6’ in both the *Plesiadapis* and *Pelycodus* figures. Though this is not an explicit statement that the central cusp within lagomorphs is the protocone, it implies that Major thought the central cusp to be homologous with the protocone in primates.

**Figure 3:**
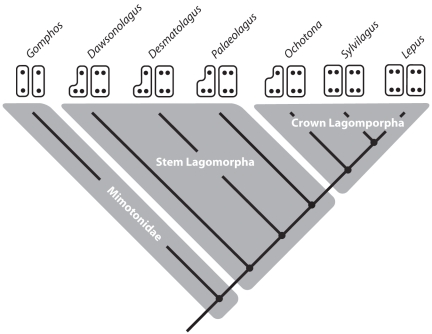
Phylogeny of Duplicidentata based on Meng et al. [**22**], Asher et al. [**19**], and Wible [**34**]. This work shows that a monophyletic Lagomorpha is nested within a paraphyletic Mimotonidae (here represented by *Gomphos*). Within Lagomorpha, *Dawsonolagus, Desmatolagus* and *Palaeolagus* represent stem-lagomorphs, within which *Sylvilagus, Lepus*, and *Ochotona* represent a monophyletic crown Lagomorpha. Each taxon has been associated a pair of schematics showing the presence of primary cusps in P^3^ (left) and P^4^ (right). In each schematic pair, buccal is up, mesial is to the right, and black dots represent cusps. In a four-cusped tooth (e.g. *Lepus* for both premolars), the upper left cusp is the paracone followed clockwise by the metacone, hypocone, and protocone. Note that the paracone is missing in the P^3^s of all taxa except *Sylvilagus* and *Lepus*. Gomphos only shows a paracone and protocone on each of its premolars.

Although several authors developed tritubercular/tubercular-sectorial theory decades earlier (e.g., [26]–[27]), Osborn [4] first applied the theory across Mammalia more broadly. In his treatment of lagomorphs Osborn [4] largely agreed with Major's [3] homology of duplicidentate molar cusps with those of *Pelycodus*, implying that the central cusp is the protocone. The discussion is brief, and it is unfortunate that the *Titanomys* tooth figured (the 114^th^ figure of [4]) seems to be grossly mislabeled, as pointed out by Bohlin [10]. Osborn [4] did not discuss any other cusps, and only additionally figured the metacone and paracone (the paracone was incorrectly labeled as Major's cusp ‘6’). Osborn had for some time interpreted the ‘primary’ tritubercular cusp as the protocone [27]–[28], which he considered derived from the primitive reptilian unicuspid condition. His recognition of the central cusp of lagomorphs as the primary cusp was the basis for its identification as the protocone.

Ehik [29] was the first to outline a hypothesis of cusp homologies within lagomorphs in detail, for which he primarily referred to the fossil ochotonid *Titanomys*. In addition to naming the cusps, Ehik [29] came to the important conclusion that upper premolars and molars shared the same basic pattern. This was based on his recognition that the P^3^ was ‘primitive’ relative to the other upper cheek teeth, and the more complex cheek tooth morphology was derived from the primitive P^3^ condition. Ehik [29] claimed that parts of P^3^ and P^4^ are easily recognized as homologous, and that the subsequent molars share the same pattern as P^4^. This insight is important because most, but not all, researchers have considered lagomorph premolars and molars to be homologous. The problem with Ehik's [29] system is largely, then, in his interpretation of the simplified three-lobed P^3^. Osborn [4] suggested that all tritubercular teeth shared an evolutionary history with a triconodont ancestor and Ehik's [29] interpretation of the lagomorph P^3^ was based explicitly on this assumption. In simple terms, Ehik [29] homologized the central cusp of lagomorphs with the protocone, but this interpretation is flawed as he recognized the P^3^ of *Titanomys* as derived from a triconodont tooth that he hypothesized had been rotated ninety degrees, where the three primary cusps are now aligned transversely rather than longitudinally. Not only is the proposed rotation unlikely on developmental and evolutionary grounds, subsequent authors have pointed out that the primary cusp of triconodonts is now considered to the paracone [6], [12]. Nonetheless, Ehik's [29] assertion that lagomorph premolars and molars are based on the same pattern has gone largely unchallenged, with the exception of work to be discussed later [10]. Ehik's [29] work was conducted within the context of the ‘premolar analogy’ theory (see [30] for a thorough review), which makes two important points: first, the primary (and first to develop) cusp of therian mammals is the paracone (and not the protocone, as had been previously thought); and second, molars are serially homologous with premolars. While Ehik [29] clearly argued the latter point, he also suggested that although the central cusp is the protocone, lagomorph teeth still supported the ‘premolar analogy’ theory but had been modified (i.e., rotated) from that condition due to the unique demands of lagomorphs lateral mastication motion.

As the ‘premolar analogy’ debate continued, Burke [1] homologized lagomorph cusps in a way that strongly supported the theory (e.g. the central cusp was interpreted as the paracone). One of his only statements regarding his reasoning, however, was that his system was ‘…more in accordance with the relationships of various molar elements in other orders of mammals, and more in keeping with observed evolutionary trends in the cheek teeth of the group itself’ ([1]:408), which was, in part, a response to the system proposed by Ehik [29]. Despite his strong statements, Burke [1] did not discuss the ancestry of lagomorphs, nor how the tooth morphology of lagomorphs is derived from a tritiberculate ancestor. His assessment is likely influenced by his acceptance [1] of the ‘premolar analogy’ theory that recognized the primary molar cusp (or first to form) as the paracone, and not the protocone.

Wood [8] conducted an extensive survey of fossil lagomorphs, and strongly supported Ehik's [29] contention regarding the serial homology among lagomorph cheek teeth, and was primarily concerned with making homology statements for lagomorph cusps in comparison to other mammals. To this end, Wood ([8], his 115^th^ figure) identified the central cusp as the metacone, a position he continued to maintain for decades [9]. His work, however, illustrates the need to carefully read discussions concerning cusp homology assessments (in particular, [8]:351–360). Although Wood [8], [9] identifies the central cusp as the metacone, he explored the idea that only the protocone was representative of the primary tribosphenic cusps (e.g., protocone, paracone, and metacone), and rightly concludes, that if this were the case the ‘premolar analogy’ theory would not hold for lagomorphs due to the absence of the paracone. Wood [8] also recognized that premolars experienced a ‘delayed’ evolution as compared to the molars, and that this also had implications for the premolar analogy theory:

*‘In some cases, however, premolar analogy cannot be applied, because the molars had already attained their full pattern while the premolars were still undifferentiated, and when the mechanical forces of mastication brought about the convergence of the premolars to the molar pattern, the teeth developed in whatever manner the genes and the mechanics of tooth function permitted at the time, which might be entirely different in its details from that followed by the molars, though leading to a similar pattern in the end.’*

([8]:354–355)

Most pointedly, Wood [8] stated that until better fossils of ancestral lagomorphs were found, the homology of cusps would remain unclear. One must consider, then, whether Wood's [8] initial assessment (albeit tentative) of the central cusp as the metacone influenced, or was influenced by, his hypothesis that ancestral lagomorphs were derived from Condylarthra, which were considered to have a lingually shifted metacone.

Bohlin [10] presented an equally extensive discussion on the homology of the lagomorph central cusp and also concluded that it was the metacone. This conclusion, however, came with the same hesitation as did Wood's [8]. Bohlin also spent considerable time discussing the premolars, including their deciduous precursors, and came to the broad conclusion that throughout lagomorph phylogeny premolars continued to ‘progress’ until they reach a complexity seen in the molars. Interestingly, he also recognized that the progression within premolars was from posterior to anterior within individual tooth positions, and opposite in molar development. This assessment was specifically referring to the buccal expansion of the loph derived from the mesiolingual cusp, in P^3^ at least. This also led Bohlin [10] to suggest that the molars and premolars developed differently, and therefore, are not serially homologous. He reached this conclusion partially through his struggle to reconcile the evolution of the mesiobuccal portion of premolars. Bohlin's [10] perspective on the differential presence of the paracone on P^3^ and P^4^ is, at times, difficult to follow, but it's clear that he is not sure whether the reduced paracone in some P^4^s is the result of the loss of a paracone, or the failure of it to fully develop (although he leans toward the second explanation).

In a brief treatment, Tobien [2] suggested that the lagomorph central cusp might be homologized with the paracone. In this interpretation, he suggested that the area buccal to the central cusp is an expanded stylar region that consisted of three cusps. The posterior cusp is homologized with the metacone, and the mesial buccal cusp is described as connected to the paracone via a paracrista (although he does not name that mesial buccal cusp). Tobien [2] concludes that the structures of the lower cheek are easily recognized as tribosphenic structures.

Russell [7] conducted a study of unworn of *Sylvilagus* to identify a central cusp, and to compare it to the central cusp of various fossil lagomorphs. In addition to identifying the central cusp in living lagomorphs the study also pointed to pantotheres as a possible ancestor to lagomorphs. This was partially based on Russell's [7] interpretation of *Eurymylus* (or something very similar to it) as an intermediary ancestor. This was important in that although *Eurymylus* was not known to have a central cusp due to advanced wear in known specimens, it had two buccal cusps that were interpreted to be the paracone and metacone. Given that, and the relationship inferred to pantotheres, Russell [7] suggested that the central cusp of lagomorphs was the amphicone.

In the context of a cladistic analysis of Lagomorpha, McKenna [6] suggested that the central cusp of lagomorphs was the protocone. In contrast to previous workers, McKenna [6] interpreted the lagomorph protocone as buccally shifted, while an associated lingual expansion of the crown produced a pericone and hypocone. This was determined largely via comparison with anagalids, although it was not shown that anagalid upper molars had undergone any lingual expansion.

Although previous authors had considered the occlusion of upper and lower teeth to discern cusp homology [8], [10], López-Martínez [11] was the first to conduct a detailed study of wear facets within the lagomorph dentition to help interpret cusp homologies. She based her work largely on the system established by Crompton [31], and recognized many of the basic tribosphenic wear patterns in fossil and living lagomorphs. As the lower dentition is more easily interpreted, López-Martínez [11] used that in addition to wear facets to homologize the lagomorph central cusp with the metacone. This work will be discussed below.

Tong and Lei [32] gave a much more complex interpretation by suggesting that the central cusp of premolars and molars should not be homologized. They interpreted the central cusps of molars as the metacone based on its position relative to the talon, but also recognized that the cusp had shifted mesiolingually. The central cusp of the premolars was interpreted as the paracone or amphicone based on the study of P^4^s of *Mimotona*, which shows one primary buccal cusp in premolars.

Averianov [12] conducted one of the most thorough of the recent studies concerned with cusp homology among lagomorphs. In that work, the central cusp is interpreted as an evolutionary novelty, not homologous with other known tribosphenic structures. This interpretation is based, partially, on the unique mastication (with predominant lateral motion) mode within lagomorphs. Averianov [12] recognized the functional significance of the central cusp and the crescentic valley in this context, and argues that they arise due to the non-functional role of the paracone and metacone. Averianov [12] also suggested that mimotonids may represent the ancestral group to lagomorphs, and claims the central cusp and crescentic valleys are new structures within Lagomorpha as they have no precursors within Mimotonidae.

Van Valen [5], [20] has given several different interpretations of the lagomorph central cusp. In his first treatment [5], he suggested that the central cusp of lagomorphs might be the protocone (or possibly, the metacone) based on the presence of two prominent buccal cusps, interpreted as the paracone and metacone. This was determined with comparisons to the insectivore *Pseudictops* and eurymylid *Eurymylus*, both of which he considered close to the ancestry of lagomorphs. This interpretation requires a significant buccal shift of the protocone, driven by the development of unilateral hypsodonty within lagomorphs. Van Valen [20] later suggested that the central cusp of lagomorphs could be the metaconule based on the presence of an enlarged metaconule in *Mimotona*. This interpretation was unique, and Van Valen [20] went on to state that additional transitional fossils would be needed to support this hypothesis.

Meng and Hu ([33]: 264) made the following description of the M^1^ of *Gobiolagus major*, “The inner surface of the fossette is covered with thin enamel. Lateral to the fossette [portion of hypostria], a widening of the metaloph indicates a transversely elongated metaconule…” While this statement interprets the metaconule as present within *Gobiolagus*, no broader discussion was given with regard to interpretation of the central cusp among all lagomorphs.

## Methods

### Establishing a phylogenetic framework and taxa used

The phylogenetic framework used here is based on the work of Meng et al. [22], Asher et al. [19], and Wible [34] (Fig. 3), which have shown that mimotonids are the ancestral group from which lagomorphs evolved. Mimotonids are known exclusively from the Late Paleocene – Middle Eocene of Central Asia, and represent a paraphyletic group that includes a nested monophyletic Lagomorpha. The tooth morphology of *Gomphos elkema*, the best-known fossil mimotonid [19], will be discussed in detail. *Dawsonolagus, Desmatolagus*, and *Palaeolagus* will be used to represent the transitional morphology of stem lagomorphs, found immediately outside crown lagomorphs. *Desmatolagus* is known from the Late Eocene – Late Oligocene of Asia and North America, and *Palaeolagus* is known from the Late Eocene – Oligocene of North America. Although these taxa show similarities with respect to their tooth crown morphology, they are markedly different in regard to features such as hypsodonty: *Palaeolagus* is fully hypseledont, whereas *Desmatolagus* retains substantial unilateral hypsodonty. *Dawsonolagus* is a recently described stem lagomorph from the Middle Eocene of China [35]. The extant taxa, *Lepus*, *Sylvilagus*, and *Ochotona* are used to represent crown lagomorphs.

A major focus of this work was deciphering cusp occlusion in mimotonids via new specimens of *Gomphos elkema* (including those from [19]) from Central Asia, including multiple individuals that include associated upper and lower dentitions that are useful for understanding the occlusion of the upper and lower cheek. In particular, specimens MAE BU 14524, 14425, and 14426 each consist of complete lower and upper tooth rows that articulate and allow for determination of the occlusion of dentitions in resting position.

## Results

### Mimotonid molars: occlusion, wear facets, and the central cusp

Wear facets have been shown to be an excellent tool for deciphering cusp homology among mammals [36]. The descriptions of facets here are based on three primary texts, Crompton [31], Butler [37], and López-Martínez [11]. In the first, wear facets are described for Mesozoic mammals and compared to modern marsupials to better understand the origin and homology of tribosphenic teeth. Although the teeth of basal duplicidentates can easily be correlated to the tribosphenic type, several derived conditions within Duplicidentata differ significantly from the primitive tribosphenic condition and require careful study. These include the loss of a stylar shelf and the expansion of the distal portion of upper teeth to include a significant post cingulum/hypocone. Butler [37] specifically dealt with taxa that exhibited similar features, such as primates and rodents, and their functional and homological implications, but used a different wear facet nomenclature. López-Martínez [11] studied the wear facets of lagomorphs and their ancestors, using the nomenclature of Crompton [36], which we follow here, while incorporating additional facets for novel morphology (e.g. facet 7).

**Figure 4:**
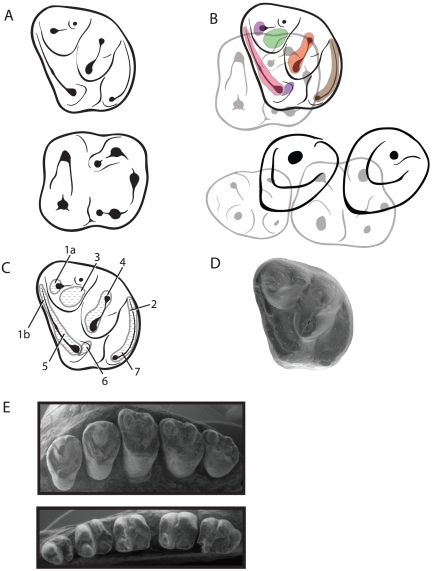
Drawings and SEM images of cheek teeth of *Gomphos elkema*. A, upper and lower molar teeth line drawings showing major cusps and cingula. Upper molar (above), lower molar (below), buccal to top, mesial to left in both teeth. B. Top, upper and lower molars in occlusion, buccal to top, mesial to left. Bottom, upper P^3^ and P^4^ premolars shown in occlusion with lower premolars, same orientations as molars. C, Major wear facets numbers of Crompton [31] shown on upper molar of *Gomphos elkema*; wear facet 7 from López-Martínez [11]. D, SEM of MAE BU 14559, M^2^ of *Gomphos elkema* for reference. E. Upper (P^3^–M^3^) and lower (P_3_–M_3_) teeth of *Gomphos elkema* (MAE BU 14426). Buccal to top, mesial to left in both tooth rows.

In general, mimotonid molar teeth share all of the primary wear facets of Crompton [36], as illustrated in figure 4. Due to the predominance of lateral motion within duplicidentates, the teeth are characterized by having several prominent lateral wear facets. The entire mesial width of upper molars becomes a wear facet as the protocone and the connected precingulum are worn against the distal wall of the trigonid of the corresponding lower tooth (combined facets 1 and 5). Although Butler [37] and López-Martínez [11] recognized these facet in lagomorphs, the condition in mimotonids is more similar to the pattern described by Crompton [31], particularly where the buccal aspect of this feature is separated into two distinct facets, one high on the paracone (facet 1a) and one along the buccal extent of the precingulum (facet 1b). As a functional unit, these facets wear the distal border of the corresponding trigonid and mesiolingual portion of the talonid basin. *Gomphos*, as in other tribosphenic teeth, also have an additional wear facet 6 that projects distobuccally from the protocone and occludes with the area of the entoconid on the corresponding lower tooth. This feature is minor in molar teeth that have a hypocone, but more prominent in premolar teeth where the hypocone is absent. With wear, an additional facet emerges on the lingual side of the paracone that occludes with the area mesial to the hypoconid (facet 3). These relationships are consistent with the interpretation of the mesiolingual cusp in the molars, or the isolated lingual cusp in the premolars, as the protocone. The mesiobuccal cusp of molars is recognized as the paracone (the isolated buccal cusp on the premolars is discussed below). In occlusion, the protocone rests within the mesiolingual portion of the talonid basin of the corresponding lower tooth, in both molars and premolars. The paracone (in molars) also wears against the mesoconid and part of the hypoconid, as evidenced by wear facet 3.

Topologically, the central cusp of mimotonid molars should be considered the metaconule based on its position relative to the metacone and its connection with that cusp via the saddled postmetaconule crista. This interpretation also recognizes two clear buccal cusps that represent the paracone-metacone complex, with the absence of a stylar region. Several mimotonids also show a slight inflation along the preprotocrista that marks a minor paraconule [38]. While previous authors have considered the central cusp in mimotonids the metaconule [39], that interpretation has not been applied to the ‘central cusp’ of stem or crown lagomorphs (although see discussion below of [20]). Our interpretation of the lagomorph cusp as the metaconule is logically based on cusp positions, albeit remarkable in that in recognizes such an enlarged metaconule — roughly equal in size to the paracone, metacone, and protocone. While the size is atypical, considering this cusp the metacone, as previous authors have done for lagomorphs, would also necessitate invoking the presence of an abnormally large cusp somewhere else on the crown. We argue that the simplest interpretation, based on topology, is that the central cusp within mimotonids is the metaconule.

In *Gomphos*, the metaconule also exhibits wear facets that are consistent with those of earlier tribosphenic mammals, although one major difference is observed with regard to occlusion. The metaconule occludes in resting position within the distobuccal portion of the talonid basin (Fig. 4). More precisely, a minor saddle occurs along the distal border of the talonid between the hypoconulid and the hypoconid on which the premetaconule crista rests. This relationship is in contrast to nearly all other tribosphenic mammals, where the metaconule occludes just outside the talonid basin between the hypoconid-hypoconulid. While this is atypical, it is functionally necessary to compensate for an enlarged, and lingually shifted, metaconule as in *Gomphos*. The hypocone, then, occludes just distal to the distolingual portion of the lower tooth, where expected. The wear facets associated with these cusps are easily reconciled with the tribosphenic pattern, such that a prominent wear facet 4 is present along the metacone-metaconule complex that wears much of the distobuccal area of the talonids basin. As shown by Crompton [31] for tribosphenic mammals, the facet is found along the mesial slope of this crista. Crompton [31] also shows that the facet is primarily found between the metacone and metaconule, as is the condition in *Gomphos*.

In this interpretation, the distal portion of the molars is composed of a hypocone and a postcingulum that has expanded to the buccal margin of the tooth. The postcingulum includes a wear facet that is partially homologous with facet 2 of Crompton [31]. The facet extends, however, to the lingual portion of the tooth and wears against the anterior position of the trigonid of the next sequentially lower molar and the distal portion of the talonid of the next molar mesially. While the buccal portion of the upper molar tooth is homologous to wear facet 2, the lingual portion is homologous to López-Martínez ' [11] wear facet 7. Within rodents, Butler [37] recognized facets 1 and 5 along the distal length of the upper molars in sciurids, which have a hypocone, but the nomenclature of López-Martínez [11] is adopted here for mimotonids.

### Duplicidentate premolars: the molarization of premolars and the trouble with cusp homology

The deciduous premolars of *Gomphos* (particularly specimen MAE BU 14425) are easily recognizable as tribosphenic teeth, being generally similar to molar teeth. In contrast, the permanent premolars of mimotonids are not easily homologized to structures in the tribosphenic system. The permanent premolars of *Gomphos* show one dominant lingual cusp and one buccal cusp (Fig. 4). The most widely, and near universally, accepted interpretation of such dual cusps in premolars in early mammals such as zalambdalestids [40] and basal eutherians [41] is that they represent the protocone and paracone. The interpretation of the protocone is easily supported by examining the occlusal relationships of premolars within *Gomphos*, which show that the lingual cusp occludes precisely within the talonid basin of the corresponding lower premolars (Fig. 4). Based on paleontological evidence, particularly with those of zalambdalestids and basal eutherians, the likely ancestors to duplicidentates [19], the buccal cusp would be considered the paracone within mimotonids.

The occlusal relationships of the buccal cusp of *Gomphos*, however, calls into question such an interpretation as the cusp occludes precisely where (and presumably functions as) the metacone based on Crompton [31], and as seen in the molars of *Gomphos*. Specifically, this cusp occludes outside the talonid basin, between the hypoconid and hypoconulid (Fig. 4). A second line of evidence for interpretation as the metacone is the position of the buccal cusp relative to the lingual cusp. With respect to the mesiodistal axis of the tooth row, the buccal cusp is found significantly distal to the protocone, approximating the exact relative position of the metacone and protocone observed in molars. This interpretation, which suggests a distal shift of the ‘ancestral’ paracone, is also consistent with the transformation seen in the lower premolars where the talonids are shorter then those of the molars due to the absence of a mesostylid and mesoconid.

If the original buccal cusp of mimotonids is, indeed, a distally shifted paracone, which becomes a metacone, this suggests that the second buccal cusp of lagomorphs has been added mesially to the primary buccal cusp. Although we will discuss cusp homology among stem lagomorphs later, it is important to look at these taxa now in regard to the evidence they show that supports a second buccal cusp is added mesially within duplicidentates. Within the crown group, the third and fourth premolars of leporids are fully molariform in that they exhibit two buccal cusps and two lingual cusps. Ochotonids, however, have a more simplified ‘J’ shaped P^3^ in which only one buccal cusp is present, while P^4^ is fully molariform. Under our proposed model, ochotonids P^3^s have retained the primitive condition in which a second mesiobuccal cusp has not been acquired; yet a second lingual cusp (hypocone) has been.

**Figure 5:**
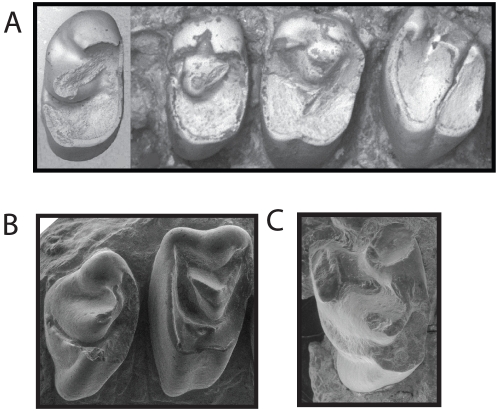
SEMs of fossil duplicidentates that show the presence (sometimes, incipiently) and absence of the paracone in premolars. All teeth shown buccal up, mesial to the left. A, P^3^ (IVPP V7499.2) and P^3^–M^2^ (IVPP 7462) of *Dawsonolagus antiquus* showing the increased development of the second buccal cusp in the distal dentition. B, P^3^ and P^4^ of *Desmatolagus gobiensis* (AMNH 83703) showing the absence of a paracone on P^3^, but presence on P^4^. C, and Isolated P^3^ of *D. gobiensis* (AMNH 83689) showing a minor, atypical, mesiobuccal cusp in the position of the paracone.

A newly described stem lagomorph, *Dawsonolagus*, gives important insights into the evolutionary transition of premolars within duplicidentates [35]. The P^4^ of *Dawsonolagus* shows two buccal cusps; the typical central cusp of lagomorphs and a wide lingual region that implies the presence of both a protocone and hypocone shelf (Fig. 5). The buccal cusps of this tooth differ in size, where the distal cusp is stronger then the mesial cusp. This is in contrast to the P^3^ of *Dawsonolagus*, which shows a single buccal cusp that is positioned on the distal margin of the tooth, generally similar to the ‘J’ shaped tooth of ochotonids and many stem lagomorphs. The distal position of the isolated buccal cusp of ochotonids and *Dawsonolagus* in P^3^ also supports its evolutionary derivation from the isolated buccal cusp of mimotonids.

This premolar cusp pattern is repeated throughout stem lagomorphs, and *Desmatolagus gobiensis* gives further insights into how and when a secondary buccal cusp may have been added to premolars. The systematic placement of *Desmatolagus* has long been problematic, where some authors have considered it leporid [8], [42]–[44], an ochotonid [44], [45], and some have placed species currently within the genus in both families simultaneously [46]. These problems are more easily understood when it is recognized that *Desmatolagus* fits phylogenetically just before the split of crown Lagomorpha [47]. As is typical for stem lagomorphs, *D. gobiensis* has a fully molariform P^4^, while its P^3^ has only one buccal cusp (Fig. 5), similar to the condition in extant ochotonids and earlier stem lagomorphs (Fig. 3). A survey of the AMNH collections has revealed several specimens that show a minor second buccal cusp that is mesial to the primary cusp (Fig. 5). Although rare, these occurrences show that a second buccal cusp is incipiently present in *Desmatolagus gobiensis* P^3^s and supports the notion that a mesial cusp is added to an isolated buccal cusp evolutionarily.

The hypothesis that a mesial cusp was added to an isolated paracone, however, creates a nomenclatural conflict. Given that the primary buccal cusp of mimotonids evolved from the paracone of basal eutherians, our interpretation suggests that the distal buccal cusp in living and extinct lagomorphs should be considered the paracone, and the ‘secondary’ buccal cusp (or that added mesially) is either a novel cusp, or another known tribosphenic cusp (e.g., parastyle). This is problematic in the context of topological nomenclature, which we support here, because it is clear that the primary buccal cusp shifts distally and becomes the metacone in both position and function.

**Figure 6:**
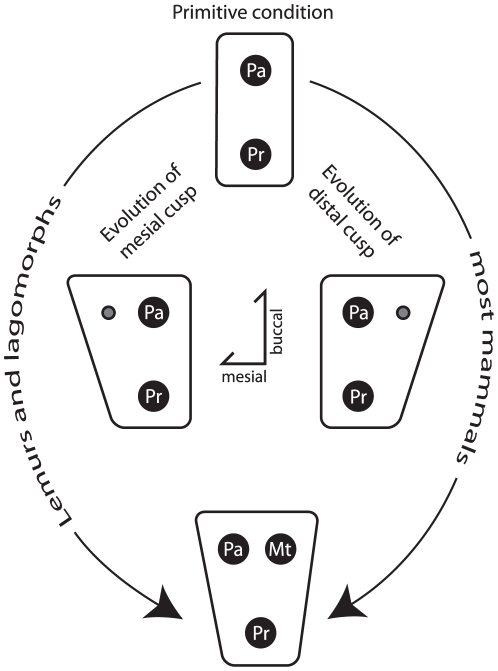
Schematic showing two ways in which a second buccal cusp can be added evolutionarily to a ‘primitive’ premolar. The primitive condition (top) shows one primary buccal cusp and one primary lingual cusp, as in *Gomphos*. The path to the right shows the addition of a second distal buccal cusp (gray), as is thought to be typical for mammals. To the left, however, the addition of a second mesial buccal cusp (gray) is shown, as described within lemurs [48] and in this study. **Pa**  =  paracone; **Pr**  =  protocone; **Mt**  =  metacone.

Another alternative would be to name the isolated (i.e. primary) buccal cusp in duplicidentates the metacone. One could argue that this scenario suggests that the isolated buccal cusp of earlier eutherians should be renamed the metacone as well. We argue that this approach unnecessarily complicates the nomenclature, and is likely to confuse cusp homologies among premolars of earlier taxa. Ultimately, the situation is complicated because the evolution of buccal cusps within duplicidentates seems to be atypical from most other mammal lineages. Tribosphenic placental ancestors have an isolated buccal cusp, considered the paracone, and give rise to many lineages in which a second buccal cusp is added distally, called the metacone. Here we show that in rare instances, a second cusp is added mesially (as in duplicidentates), but that the secondary cusp functions exactly as the paracone, whereas the primary cusp has likely undergone a distal shift (relative to the protocone) and had begun to function as the metacone (Fig. 6). In essence, a paracone becomes a metacone.

Taking both historical interpretations of the paracone and topology into account, we propose a third alternative solution. We now know that the buccal cusp in the premolars of mimotonids should be considered the paracone based on the its clear evolution from a paracone in earlier lineages, but that a secondary buccal cusp is added mesially as premolars become molarized within stem lagomorphs (Fig. 6). We argue here that this newly acquired second mesial cusp should then be considered the paracone (which is functionally and topologically supported), and the ‘original’ buccal cusp in mimotonids should then be considered the metacone. This evolutionary scenario is nearly identical to the condition observed in the premolars of some lemurs [48], which the authors described as a *discontinuity in cusp homology*. In that study, Jernvall et al. [48] show convincingly that some bamboo lemurs (*Hapalemur*) exhibit premolars that are transitionally becoming molariform by adding a mesial cusp to the previously dominant isolated buccal cusp. As Jernvall et al. [48] also point out, both Van Valen [30] and Butler [49] showed that a similar discontinuity exists in the evolution of perissodactyl premolars where a second lingual cusp has been added mesially to a purported protocone in several lineages on the P^3^, where typically it has been viewed that a hypocone is added distally to a protocone. Combined, these studies suggest that, although rare, certain lineages add a second cusp mesially to the paracone or protocone, and that the fossil record does not support the assumption that secondary cusps are always added distally. While we agree that the scenario described in these lemurs closely matches that observed here in mimotonids, we believe that it is not a *discontinuity in homology*, as described by Jernvall et al. [48], but rather the unique character transformation seen in these lineages calls for a *discontinuity in nomenclature*. The homology itself, or which cusp becomes which, is nicely demonstrated at the population level by Jernvall et al. [48], and strongly reinforced via our example from the fossil record. The primary question that remains is how should cusp nomenclature best reconcile an increasingly better understanding of cusp evolution with a modern understanding of the plasticity of developmental systems that form these cusps? Or more bluntly, should we expect cusp nomenclature to reconcile these issues?

To this end, we outline here the details of this third alternative, that a nomenclatural shift should takes place similar to what Jernvall et al. [48] suggested for lemurs. Our argument is grounded in the assumption that *cusp nomenclature should be strictly a topological consideration*. Homology is a hypothesis most-often framed within a phylogenetic tree; whereas, we argue that names should describe morphology. In this scenario, the single buccal cusp of mimotonids should continue to be referred to as the paracone because this maintains the most historical consistency with older taxa (i.e., historical congruency). Once a second buccal cusp appears, as in the P^4^ of stem lagomorphs (e.g. *Dawsonolagus*), the distal cusp should be considered as the metacone and the (newly acquired) cusp as the paracone. In addition, the isolated, single buccal cusp in P^3^ of stem lagomorphs and ochotonids should also be called the metacone when a hypocone is present, as the appearance of hypocone clearly distinguishes the isolated mesial cusp as a metacone topologically. The incipient mesially placed buccal cusp in *Desmatolagus* should also be referred as the paracone. This solution maintains meaningful nomenclature, recognizing the evolutionary history of the buccal cusp with respect to older taxa, but also takes into account that there are different evolutionary pathways that create the ‘ same cusps,’ as demonstrated by the examples lemurs [48] and horses [3], [49].

Furthermore, recent experimental and quantitative modeling work in the development of tooth cusps supports the notion that small genetic changes may have wide ranging effects on tooth cusp morphology [50], [51], [52]. A more detailed understanding of the hierarchy of tooth morphogenesis has supported a reiterative process of morphogenesis, both experimentally and quantitatively, in which gene expression begins initial cusp patterning, but cell and tissue interactions have significant consequences on the end morphology of a tooth [53], [54]. Osborn [53] recently developed a model for human tooth development, in which a wide range of tooth morphologies can be predicted from the developing tooth by changing four directional force parameters during proliferation from a single epithelial cell, mimicking the effects of cell and tissue interactions. More relevant to our study, Jernvall et. al [54] showed experimentally that, while mice and voles have nearly identical genetic control of initial cusp patterning, they exhibit different overall adult cusp positions due largely to the different position in which the second cusp in their lower first molar appears. In voles, the first and second cusps to develop are offset from one another laterally, whereas they are parallel in mice. These initial patterns have a cascade effect on the remaining cusp formation. In short, the parallel cusp patterning observed in mice seems to be the product of an initial mesial shift of their second cusp. Combined, these studies show that developmental mechanisms exist that could create the shift in cusp position observed in both duplicidentates and lemurs, and more importantly, how subsequent cusps could form around a shifted cusp in what is a typical tribosphenic pattern. A nomenclatural system must compensate for this dynamic ‘interplay between molecular signaling and tissue growth’ [48].

### The central cusp in Lagomorpha

**Figure 7:**
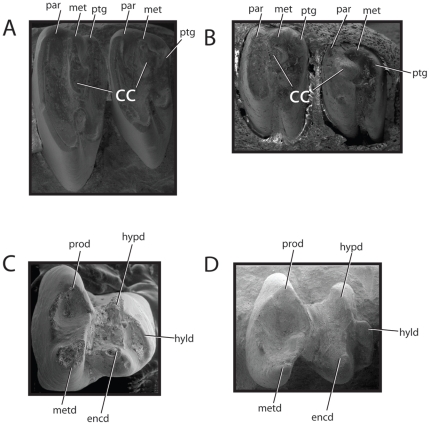
SEMs showing slightly worn upper molars and lower P_4_s. A, M^1–2^ of *Desmatolagus gobiensis* (AMNH 19106). Note cusp names and tribosphenic terms for the tripartite structure of the buccal margin of the teeth. Mesial to left, buccal to top. B, M^1–2^ of *Lepus californicus* (AMNH 5887:Mammalogy). Note cusp names and tribosphenic terms for the tripartite structure of the buccal margin of the teeth. Mesial to left, buccal to top. C, P^4^ of *D. gobiensis*, mesial to left, buccal to bottom. D, P^4^ of *Palaeolagus haydeni*, mesial to left, buccal to bottom.

No structure has confounded the homology of lagomorph teeth more than the central cusp. As discussed above, the centrally located cusp of mimotonids correspond to the metaconule and recent phylogenetic results show that lagomorphs were derived from a mimotonids stock [19], [22], [34]. We therefore conclude that the central cusp of stem and early crown lagomorphs should also be considered the metaconule, based on the topographic position of the cusp and its connection to the metacone via the premetaconule crista (Fig. 7, but see also figs. 1 and 2).

This interpretation is at odds with the identification of the central cusp by López-Martínez [11], who identified the wear facet along the mesial side of the central cusp extending into the buccal border of the tooth as wear facet 4 (i.e., the mesial wear facet of the metacone). In this interpretation, this wear facet would extend from the “metacone” buccally to an unnamed cusp. Crompton [31], however, recognized that such a facet extends from the metaconule to the metacone in tribosphenic mammals. Given the similarity to facet patterns and overall topology of cusps with mimotonids, it is more plausible to interpret the central cusp of lagomorphs as the metaconule, rather then to invoke a significant lingual shift in the metacone and the appearance of a novel cusp buccally, as López-Martínez suggests [11].

Some confusion has also been the result of the position of the *central* cusp. While the cusp is centrally located within the crowns of mimotonids and lagomorphs, the cusp is positioned at the distal border of the trigon, as formed by the primary tribosphenic cusps, and only appears in the central portion of the crown due to a significant expansion of the hypocone/postcingulum. Understanding this relationship also allows us to draw important conclusions regarding the buccal border of cheek teeth in stem lagomorphs. As is typical with wear, a tripartite structure is found along the buccal margin of lagomorph check teeth (Fig. 7). These structures have been problematic to interpret (or usually not recognized) as authors have been unclear how the buccal lophs are related to tribosphenic cusps. The three lophs represent the paracone, metacone, and buccal extension of the post cingulum, respectively, mesially to distally. This pattern is repeated throughout stem- and crown lagomorphs and is easily resolved with our interpretation of lagomorph tooth cusps. We, therefore, argue that the central cusp of lagomorphs is positioned topologically where the metaconule should be, functions as a metaconule with regard to wear facets (albeit, as discussed, has a slightly different occlusal position), and should be considered homologous with the metaconule in all living and extinct lagomorphs.

### The crescentic valley

Once the homology of the central cusp has been established, it is then possible to understand the crescentic valley (Fig. 1) in light of its tribosphenic origin. The central cusp is found just buccal to the apex of the valley, and two prominent wings of the valley project mesially and distally to the buccal portion of the crown. The distal wing of the crescentic valley is here interpreted as the talon basin as it serves to separate the postcingulum from the metaconule/premetaconule crista. Indeed, with wear, the postcingulum is sometimes completely separated from the rest of the crown (Fig. 5, see *Dawsonolagus*). The mesial wing is the trigon basin (as also recognized by Meng and Hu [33]), found largely between the protocone and paracone.

Previous workers have suggested that the central cusp and the crescentic valley disappear evolutionarily in more derived lagomorph taxa, particularly among living members of the crown group. Several studies have pointed out, however, that the central cusp and crescentic valleys are present, albeit for an ontogenetically brief period, within living ([7], [15]; this study, fig. 7) and recently extinct [14] lagomorphs. The latter study shows a remarkable similarity among crown patterns in living lagomorphs to those of even the earliest duplicidentates, including the tripartite buccal structure determined to the be the paracone, metacone, and post cingulum.

### Lower dentition

The lower dentition of duplicidentates is more ‘simple,’ and the tribosphenic nomenclature is more easily applied. The strongest trend in the lower teeth is the loss of a paraconid, a condition that occurs in many other mammalian groups, particularly rodent lineages. The simple two-lophed teeth of adult lagomorphs make it difficult to discern typical tribosphenic structures. As with upper teeth, however, once newly erupted and unworn teeth are studied, it becomes clear that the lower teeth of lagomorphs are typically tribosphenic. Isolated P_4_s of *Desmatolagus* and *Palaeolagus* (Fig. 7) show a clear trigonid basin bordered by a pronounced metaconid and protoconid. Two strong cusps, the hypoconid and entoconid, are distinguished on the talonid. At the distal end of the talonid, the hypoconulid has transformed from a cusp to a loph. As pointed out in mimotonids, particularly within the molars, the mesial portion of the talonids has expanded to compensate for a mesoconid and mesostylid.

This study does not deal directly with the lower third premolar of lagomorphs, the evolution of which has played a significant role in the systematics of the group (see [55], for a summary). In contrast to other lower teeth, the P_3_ seems to have undergone substantial evolution within the crown group, largely via the addition of several lophate structures. In that respect, much of this newly derived morphology are novel structures, and in our opinion, are not easily, or appropriately, identified as tribosphenic structures. Primitively, as indicated by mimotonids, the P^3^ appears to be a simplified trigonid and talonid, with four prominent cusps (protoconid, metaconid, hypoconid, and entoconid). Stem lagomorphs then simplify their P_3_s, primarily through the reduction of the lingual portion of the trigonid, and loss of the metaconid. This gives the overall ‘C’ shape that is typical for early lagomorphs. Many workers have discussed the evolution of lagomorph P_3_, particularly Dice [56] and Wood [8], and we recommend similar terminology be used due to the complex evolution history of this tooth; we suggest that readers refer to [57] for P_3_ nomenclature.

## Discussion

The nomenclatural system outlined here (Fig. 2) is based on a highly resolved phylogenetic framework and the description of a series of fossils that show a clear evolutionary transition from a tribosphenic tooth type to the simplified bilophodont teeth of crown lagomorphs. In addition to important insights regarding the proper homology of duplicidentate cusps, we are able to outline several broad evolutionary trends in the evolution of duplicidentate dentitions. Most obvious among these is that duplicidentate cheek teeth are distinguished from more typical tribosphenic teeth by the loss of a stylar region and the development of a strong hypocone/post cingulum, first in molars, later in premolars. Mimotonids also show distinct differences between premolars and molars. Within their lower tooth row, their talonids (primarily within premolars) are generally shorter anteriorly via the absence of the mesostylid and mesoconid. The correlative condition in upper teeth is the absence of multiple cusps on the buccal margin of the premolars — with no cusp that *functions* as the tribosphenic paracone within mimotonids. While the absence of the mesostylids and mesoconids is maintained in lagomorphs (and the condition is subsequently acquired in the molars), the appearance of a second buccal upper cusp in premolars has a complicated history, atypical for mammals. The second buccal cusp initially occurs within P^4^, and later in P^3^ (leporids); however, certain lagomorphs (ochotonids) never develop a second buccal cusp in P^3^. Both Wood [8] and Bohlin [10] recognized delayed evolution of premolars relative to molars in the fossil record of lagomorphs.

Because the isolated buccal cusp of mimotonids functions as a metacone rather then a paracone, we propose a *discontinuity in cusp nomenclature* for duplicidentates, similar to that described in lemurs [48], where the isolated buccal cusp of mimotonids should be recognized as the paracone, but after appearance of a second buccal cusp, or a hypocone, the original buccal cusp should then be called the metacone. While this is conceptually complicated, the resultant system allows for the best resolution of the historical constraint of cusp terminology, recognizing that the buccal cusp within mimotonids evolved from the paracone of earlier branching ancestral mammals. Unlike most mammals, however, the original buccal cusp of lagomorphs has been shifted distally and becomes the metacone functionally. Our system considers the unique evolutionary history of each buccal cusp, while embracing the topological considerations of homology among lagomorphs as compared to other mammals. In short, we call it the metacone when it becomes the metacone topologically. Most importantly, however, this system recognizes the dynamic, and often disparate, developmental processes that may lead to the appearance of a new cusp. In short, our argument is that it is much more easy to discern topology of form then the underlying developmental and molecular processes that produce form, and therefore, a nomenclature based on topology will be much more stable, and in turn, more useful.

Based on the study of mimotonid material, it is clear that the prominent central cusp is homologous with the metaconule. Previous workers had recognized this [39], but only Van Valen [20] and Meng and Hu [33] had suggested that the central cusp of lagomorphs might also be homologous with the metaconule. In his discussion of the lagomorph central cusp, Van Valen [20] states that both Averianov [12] and Meng and Wyss [47] suggest the central cusp of lagomorphs is the metacone; however, Averianov [12] clearly stated that the lagomorph central cusp was not homologous with the primary cusps of Eutheria (i.e. protocone, paracone, or metacone), and that the central cusp was an evolutionary novelty. Despite this confusion, it's clear that both Averianov [12] and Van Valen [20] recognized that the protocone is the mesiolingual cusp within lagomorphs and that the two buccal cusps are the paracone and metacone, but differ in their assessment of the central cusp.

Van Valen [20] suggested that an intermediary between *Mimotona* and lagomorphs is needed to clearly show the central cusp of lagomorphs is indeed the metaconule. While phylogenetic studies do not clearly show that *Gomphos* is this intermediary, a prominent metaconule is pervasive throughout Mimotonidae. Our work presented here, as well as that summarized by others, shows that the central cusp of lagomorphs functions as the metaconule in lagomorphs, and is topologically consistent with that interpretation given other prominent tribosphenic structures.

Averianov [20] conducted the last study to deal specifically with cusp homology among lagomorphs, where he used occlusal patterns to suggest that the central cusp of lagomorphs was a novel structure. We agree with Averianov's [12] assessment that the central cusp is not one of the primary tribosphenic cusps (i.e., the protocone, paracone, or metacone), but do not think that this implies that the central cusp is an entirely new feature. Averianov [12] suggested that mimotonids do not have crescentic valley, and implies that this means the feature is newly evolved in lagomorphs. Given the clear relationship between mimotonids and lagomorphs, the mimotonid metaconule is available, and almost certainly evolved into the central cusp of lagomorphs. As we have shown here, the crescentic valley is also present within Mimotonidae in the form of the talonid and trigonid basins, but has not deepened yet to form the crescentic valley of Lagomorpha. The strong trend in increased unilaterally hypsodonty (leading to hypseledonty) within Duplicidentata facilitates the deepening of the talon and trigon basins to form a crescentic valley, and ultimately, the shallowing of this valley in living lagomorphs.

Several authors, in particular Averianov [12], have given thorough discussions as to *why* lagomorphs have developed a central cusp. The question should now be similarly asked with regard to mimotonids; or more precisely, why have duplicidentates enlarged what is typically a minor cusp, the metaconule? As others have pointed out, lagomorph mastication differs from most other mammals in that their power stroke is in the lateral direction. This is evidenced by the jaw musculature in living leporids, which have a reduced *temporalis m*. (primary crushing muscle) and an enlarged *pterygoid m*. (primarily involved in lateral jaw motion) [22]. This is in contrast to rodents, whose power stroke is most typically propalinal. As is well documented within rodents, many groups develop lophate teeth that serve to maximize the mastication efficiency via increasing enamel surfaces. In many ways, the metaconule and associated hypostriae serve a similar purpose for duplicidentates. The benefit of enlarging the metaconule is clear, but more importantly, the occlusion of the metaconule within the talonid basin along with the protocone has the additional advantage of making the lateral stroke more useful by putting more prominent cusps in direct contact with the talonid basin during a greater duration of the chewing motion. This is also the case for the deepening of the talon and trigon basins (i.e. crescentic valley). More derived lagomorphs also develop lingual hypostriae that increase the wear capacity of teeth, and the reduction of the depth of the crescentic valley is correlated with expansion of the hypostria [13]; both of which serve the same function. All of these structures, the metaconule, crescentic valley, and hypostria, are aligned so that they increase the efficiency of the lateral power stroke of duplicidentates. Their presence also highlights an important evolutionary trend within duplicidentates that is tightly correlated with the development of unilateral hypsodonty; that is, in evolutionary sequence, the enlargement of the metaconule, deepening of the crescentic valley, and finally, the development of a lingual hypostria. The metaconule's increased utility is the product of its increased size, whereas the development of the crescentic valley and the lingual hypostria necessitate increased height of the tooth column. This is accomplished initially by an increase in unilateral hypsodonty, and ultimately, by the evolution of hypseledonty in lagomorphs.

It is also important to revisit the work of Ehik [29], who was the first to suggest that there is serial homology among lagomorph cheek teeth. Topological comparisons strongly support this, as the teeth are nearly identical in crown lagomorphs. The only difference is the degree of molarization of the premolar. It now is clear that the ‘J’ pattern of some fossil and extant lagomorph P^3^s is the result of the lack of development of a secondary buccal cusp, and the variable buccal expansion of the preprotocrista. We show that the evolutionary history of duplicidentates includes the delayed evolution of a secondary buccal cusp in premolars, as further supported by the incipient presence of a paracone in some stem lagomorphs. In general, Ehik [29] was correct in his assessment of the serial homology among lagomorph teeth, and his general recognition that the upper tooth row of lagomorphs shows varying degrees of molarization. More precisely, however, it's clear that the premolars and molars of duplicidentates have different evolutionary histories. It is difficult, if not impossible, to trace the history of duplicidentate molars to ancestors that do not have the primary tribosphenic cusp, and in turn, understanding the sequence of origin of those cusps in problematic. Because of this, it's unclear whether the evolutionary sequence is the same as that observed within premolars. What is certain is that the molarization of premolars happened much later in the evolutionary history of duplicidentates than it did in the molars. Wood [8] struggled with our inability to recognize whether molariform premolars and molars developed the same way, and were in turn, homologous, and his overall discussion of the evolution of lagomorph premolars was prescient:

*‘For the sake of simplicity, and in the entire absence of any evidence one way or the other, it has been assumed in the present work that the cusps of the premolars are actually homologous to those with which they appear to be homologous, but that they may not have passed through the same stages in reaching this ultimate pattern.’*

([8]:355)

This begs the question: how do we actually recognize serial homology? We can diagnose it via topology; however, its definition is more complicated than recognizing the ancestry of lineages as we do for primary homology; we must identify the underlying processes that produced the structures, and show that they are similar. At the level of teeth, it's obvious that premolars and molars are serial homologous, but the serial homology of the detailed structures (i.e. cusps) is unclear as the developmental processes that likely produced them will remain unknown. For this reason, we defer to Wood's [8] quote above.

Given the system presented here, and the recent phylogenetic studies on duplicidentates, it is useful to review some of the historical treatments of lagomorph tooth cusp homology again. It seems that many early workers were framing their ideas of the homology of the central cusp based on which tribosphenic cusp was the primary cusp. Much of the disagreement homologizing it with the protocone [3], [4] or the paracone [1], [29] revolved around the advancement of the ‘premolar analogy’ theory that shifted the focus from the protocone to the paracone. After that, Wood [8] and Bohlin [10] were the first to suggest the central cusp was the metacone. It is more important to note, however, that both of these workers presented expansive discussions regarding other homologies, and were both in strong agreement that the true homology of the central cusp would not be known until more primitive fossil lagomorphs were discovered. Bohlin [10] also gave an interesting, but brief, discussion regarding the polarity of the loss/appearance of the paracone in lagomorph premolars. He rightly concluded that, given the present data, it was unclear whether the paracone had been lost, or had yet to evolve. It now seems apparent that the premolar paracone of lagomorphs is a derived feature relative to their ancestors, the mimotonids.

We present here the first tooth cusp nomenclatural system for duplicidentates that spans all living and extinct species and is based on well-resolved phylogenies and the examination of fossils that bridge the morphological gap between tribosphenic teeth and typical lagomorph teeth. We outline the evolutionary history of several important cusps, and in turn, highlight their homologies, but argue that a nomenclatural system should not always mirror a hypothesis of homology. While we present strong evidence that shows that the central cusp of lagomorphs is the metaconule, an understanding of the homology among premolars is complicated by an evolutionary history that seems atypical as compared to other mammalian groups. We are confident in our assessment of evolutionary history of premolar cusps, particularly the buccal cusps, but we present a nomenclatural system that serves to be functionally useful, yet also recognizes the complex history of premolars cusps. The evolution of buccal cusp within duplicidentates also shows that the tribosphenic pattern has evolved in several ways. We refrain from introducing new terminology for the P_3_s of lagomorphs, as it is apparent that, although the tooth is derived from a simple tribosphenic precursor, the complexity observed within crown lagomorphs is highly apomorphic and not reasonably homologized with tribosphenic structures.

## References

[1] Burke JJ (1934). *Mytonolagus*, a new leporine genus from the Uinta Eocene series in Utah.. Annals of the Carnegie Museum.

[2] Tobien H (1974). Zur Gebissstruktur, Systematik und Evolution der Genera *Amphilagus* und *Titanomys* (Lagomorpha, Mammalia) aus einigen Vorkommen im juengeren Tertiär Mittel- und Westeuropas. [The tooth structure, systematics and evolution of the genera *Amphilagus* and *Titanomys* (Lagomorpha, Mammalia) from some localities in the later Tertiary of middle- and western Europe].. Mainzer Geowissenschaftliche Mitteilungen.

[3] Major CIF (1899). On fossil and Recent Lagomorpha.. Transactions of the Linnean Society of London.

[4] Osborn HF (1907). Evolution of mammalian molar teeth..

[5] Van Valen L (1964). A Possible Origin for Rabbits.. Evolution.

[6] McKenna MC (1982). Lagomorph interrelationships.. Geobios, mémoire spécial.

[7] Russell LS (1958). The dentition of rabbits and the origin of Lagomorpha.. National Museum of Canada, Bulletin.

[8] Wood AE (1940). The mammalian fauna of the White River Oligocene: Part III Lagomorpha.. Transactions of the American Philosophical Society.

[9] Wood AE (1957). What, if anything, is a rabbit?. Evolution.

[10] Bohlin B (1942). The fossil mammals from the Tertiary deposit of Taben-buluk, western Kansu. Part I: Insectivora and Lagomorpha.. Palaeontologia Sinica, New Series C.

[11] López-Martínez N, Luckett WP, Hartenberger JL (1985). Reconstruction of ancestral cranioskeletal features in the order Lagomorpha.. Evolutionary relationships among rodents.

[12] Averianov A (1998). Taxonomic notes on some recently described Eocene Glires (Mammalia).. Zoosystematica Rossica.

[13] Sych L (1977). Evolutionary trends in dentition of Lagomorpha.. Acta Zoologica Cracoviensia.

[14] Sych L (1967). Unworn teeth of *Hypolagus brachygnathus* Kormos (Leporidae, Mammalia).. Acta Zoologica Cracoviensia.

[15] Sych L, Sych B (1976). An evolutionary interpretation of several ontogenetic stages of the tooth development in the rabbit.. Acta Zoologica Cracoviensia.

[16] Sych L (1975). Lagomorpha from the Oligocene of Mongolia. In Kielan-Jaworowska Z, ed. Results of the Polish-Mongolian Paleontological Expeditions, Part 6.. Palaeontol. Polonica.

[17] Tobien H (1975). Zur Gebißstruktur, Systematik und Evolution der Genera *Piezodus*, *Prolagus* und *Ptychoprolagus* (Lagomorpha, Mammalia) aus einigen Vorkommen im jungeren Tertiär Mit-tel und Westeuropas.. Notizblatt des Hessischen Landesam- tes fur Bodenforschung zu Wiesbaden.

[18] Bair A (2007). A model of wear in curved mammal teeth: controls on occlusal morphology and the evolution of hypsodonty in lagomorphs.. Paleobiology.

[19] Asher RJ, Meng J, Wible JR, McKenna MC, Rougier (2005). Stem Lagomorpha and the Antiquity of Glires.. Science.

[20] Van Valen L (2002). How did rodents and lagomorphs (Mammalia) originate?. Evolutionary Theory.

[21] Hall BK (1994). Homology: The hierarchical basis of comparative biology..

[22] Meng J, Hu Y-M, Li C-K (2003). The osteology of *Rhombomylus* (Mammalia: Glires): implications for the phylogeny and evolution of Glires.. Bulletin of the American Museum of Natural History.

[23] Simpson GG (1936). Studies of the earliest mammalian dentitions..

[24] Major CJF (1873). Nagerüberreste aus bohnerzen süddeutschlands und der schweiz nebst beiträgen zu einer vergleichenden odontographie con Ungulaten and Unguiculaten.. Palaeontographica.

[25] Major CJF (1893). On some Miocene squirrels, with remarks on the dentition and classification of the Sciurinae.. Proceedings of the Zoological Society.

[26] Cope ED (1875). On the homologies of the Sectorial Tooth of Carnivora.. Proceedings of the Academy of Natural Sciences, Philadelphia.

[27] Osborn HF (1895). The History of the Cusps of the Human Molar Teeth. [Address at the Founding of the New York Institute of Stomatology.].. International Dental Journal.

[28] Osborn HF (1904). Paleontological Evidence for the Original Tritubercular Theory.. American Journal of Science.

[29] Ehik J (1926). The right interpretation of the cheek teeth tubercles of *Titanomys*.. Annales Musei Nationalis Hungarici.

[30] Van Valen L (1982). Homology and causes.. Journal of Morphology.

[31] Crompton AW (1971). The origin of the tribosphenic molar. In: Kermack DM, Kermack KA, editors. Early Mammals.. Zoological Journal of the Linnean Society.

[32] Tong Y-s, Lei Y-z (1987). Fossil lagomorphs (Mammalia) from the Hetaoyuan Eocene of Xichuan, Henan.. Vertebrata PalAsiatica.

[33] Meng J, Hu Y-M (2004). Lagomorphs from the Yihesubu Upper Eocene of Nei Mongol (Inner Mongolia).. Vertebrata PalAsiatica.

[34] Wible J (2007). On the Cranial Osteology of the Lagomorpha.. Bulletin of Carnegie Museum of Natural History.

[35] Li C-K, Meng J, Wang, YQ (2007). *Dawsonolagus*, a primitive lagomorph from the Eocene Arshanto Formation, Inner Mongolia, China.. Bulletin of the Carnegie Museum.

[36] Bown TM, Kraus MJ, Lillegraven JA, Kielan-Jaworowska Z, Clemens WA (1979). Origin of the tribosphenic molar and metatherian and eutherian dental formulae.. Mesozoic Mammals: The First Two-thirds of Mammalian History.

[37] Butler PM, Luckett WP, Hartenberger JL (1985). Homologies of molar cusps and crests, and their bearing on assessments of rodent phylogeny.. Evolutionary Relationships Among Rodents.

[38] Meng J, Kraatz BP, Wang Y-Q, Ni X-J, Gebo DL (2009). A new species of *Gomphos* (Glires, Mammalia) from the Eocene of the Erlian Basin, Nei-Mongol, China: American Museum Novitates.

[39] Meng J, Bowen GJ, Ye J, Koch PL, Ting S-y (2004). *Gomphos elkema* (Glires, Mammalia) from the Erlian Basin: Evidence for the Early Tertiary Bumbanian Land Mammal Age in Nei-Mongol, China.. American Museum Novitates.

[40] Wible JR, Novacek MJ, Rougier GW (2004). New data on the skull and dentition in the Mongolian Late Cretaceous eutherian mammal *Zalambdalestes*.. Bulletin of the American Museum of Natural History.

[41] Wible JR, Rougier GW, Novacek MJ, Asher R (2009). The eutherian mammal *Maelestes gobiensis* from the Late Cretaceous of Mongolia and the phylogeny of Cretaceous Eutheria.. Bulletin of the American Museum of Natural History.

[42] Mathew WD, Granger W (1923). Nine new rodents from the Oligocene of Mongolia.. American Museum Novitates.

[43] Burke JJ (1936). *Ardynomys* and *Desmatolagus* in the North American Oligocene.. Annals of the Carnegie Museum.

[44] Teilhard de Chardin P (1926). Description de mammifères Tertiaire de Chine et de Mongolie.. Annales de Paleontologie.

[45] Bohlin B (1937). Oberoligozäne Saügetiere aus dem Shargaltein-Tal (Western Kansu).. Palaeontologica Sinica, new series C.

[46] Dawson MR (1967). Lagomorph history and the stratigraphic record.. University of Kansas Department of Geology Special Publication.

[47] Meng J, Wyss AR (2001). The Morphology of *Tribosphenomys* (Rodentiaformes, Mammalia): Phylogenetic Implications for Basal Glires.. Journal of Mammalian Evolution.

[48] Jernvall J, Gilbert GC, Wright PC, Fleagle JG, Gilbert CC (2008). Peculiar tooth homologies of the greater bamboo lemur (*Prolemur  =  Hapalemur simus*): When is a paracone not a paracone?.

[49] Butler PM (1952). Molarization of the premolars in the Perissodactyla.. Proceedings of the Zoological Society of London.

[50] Salazar-Ciudad I, Jernvall, J (2010). A computational model of teeth and the developmental origins of morphological variation.. Nature.

[51] Kangas AT, Evans AR, Thesleff I, Jernvall J (2004). Nonindependence of mammalian dental characters.. Nature.

[52] Jernvall J (2000). Linking development with generation of novelty in mammalian teeth.. Proceedings of the National Academy of Sciences.

[53] Osborn JW (2008). A model of growth restraints to explain the development and evolution of tooth shapes in mammals.. Journal of Theoretical Biology.

[54] Jernvall J, Keränen SVE, Thesleff I (2000). Evolutionary modification of development in mammalian teeth: Quantifying gene expression patterns and topography.. Proceedings of the National Academy of Sciences.

[55] López-Martínez N, Alves PC, Ferrand N, Hackländer K (2008). The lagomorph fossil record and the origin of the European rabbit..

[56] Dice LR (1929). The phylogeny of the Leporidae, with description of a new genus.. Journal of Mammalogy.

[57] White JA (1991). North American Leporinae (Mammalia, Lagomorpha) from Late Miocene. (Clarendonian) to Latest Pliocene (Blancan).. Journal of Vertebrate Paleontology.

