# Correction: Evolutionary Patterns in the Dentition of Duplicidentata (Mammalia) and a Novel Trend in the Molarization of Premolars

**DOI:** 10.1371/annotation/f6605c02-1a75-4bea-aff5-b79fe3585d18

**Published:** 2010-12-02

**Authors:** Brian P. Kraatz, Jin Meng, Marcelo Weksler, Chuankui Li

Information was left out of the funding section. The funding section should read: "BPK was supported by a research fellowship at the American Museum of Natural History. Funding was provided via National Science Foundation (NSF) grants DEB EF-0629811, EAR-0120727, BCS-0309800 and Major Basic Research Projects of MST of China (2006CB806400). The funders had no role in study design, data collection and analysis, decision to publish, or preparation of the manuscript."

